# The risk of suicide in patients with critical illness: A population-based study in Taiwan

**DOI:** 10.1097/MD.0000000000030656

**Published:** 2022-09-30

**Authors:** Wei-Syun Hu, Cheng-Li Lin

**Affiliations:** a School of Medicine, College of Medicine, China Medical University, Taichung, Taiwan; b Division of Cardiovascular Medicine, Department of Medicine, China Medical University Hospital, Taichung, Taiwan; c Management Office for Health Data, China Medical University Hospital, Taichung, Taiwan

**Keywords:** critical illness, sepsis, septic shock, suicide

## Abstract

The authors investigated retrospectively the association between critical illness and risk of suicide attempts. The data are from Taiwan’s National Health Insurance Research Database. Propensity score matching, multivariable models, Kaplan–Meier analysis, and competing risk analysis were used to explore this association. The authors found that patients having an critical illness were associated with increased risk of suicide attempts after adjusting for risk factors (adjusted hazard ratio = 2.98; 95% confidence interval = 1.46–6.08). Among different subtypes of critical illness, patients with sepsis/septic shock exhibited the highest risk of suicide attempts (adjusted hazard ratio = 3.43, 95% confidence interval = 1.52–7.74). An association between critical illness and suicide attempts was shown. Sepsis/septic shock was found to confer the highest risk in these specific population.

## 1. Introduction

The enormous improvement in critical care is obvious in past years^[1–3]^; however, the psychologic outcomes among patient surviving critical illness obtained relatively less attention because most physicans and investigators focus more effort on the physiological mechanism rather than psychological impact.^[4,5]^ In the modern society, psychologic disorder is increasingly recognized as a huge global burden and the balance and homeostasis of body-mid or physio-psychological inter-relationship have been proposed.^[6,7]^ Indeed, survivors of intensive care are known to be at increased risk of anxiety, depression, and post-traumatic stress disorder, which is associated with increased risk of suicide.^[8,9]^ Several reports have shown an increased risk of suicide and self-harm in patients surviving from critical illnesses, acute stroke and acute myocardial infarction. In this regard, to enhance the global care for these groups who surviving critical illness, the author used a well-validated nationwide dataset to explore this issue.^[10]^ The paper describes a positive multivariate association between a diagnosis of critical illness and subsequent diagnosis of suicide using a robust database and the big sample size.

## 2. Methods

### 2.1. Data source

This retrospective population-based cohort study was designed to assess the relationship between critical illness and the risk of suicide. Data sources conducted in this present study were retrieved from claims data of Taiwan’s National Health Insurance Research Database (NHIRD), which covers approximately 99% of Taiwan residents.^[10]^ The National Health Research Institute receives insurance claims data from the National Health Insurance Administration and subsequently compiles them into NHIRD for research purposes since 1995. The NHIRD has been extensively used for epidemiological studies. The data utilized in this current study was the Longitudinal Health Insurance Database (LHID 2000), a subset of the NHIRD. The LHID 2000 consists of 1 million randomly collected samples from the NHI system in 2000, and contains historical ambulatory and inpatient care data. Diseases were classified on the basis of the International Classification of Diseases, Ninth Revision, and Clinical Modification (ICD-9-CM).^[11,12]^ The accuracy and validity of NHIRD diagnosis codes have been documented. All personal information was encrypted to preserve patient anonymity. This study has been approved by the Research Ethics Committee at China Medical University Hospital (CMUH104-REC2-115(CR-7)).

### 2.2. Study population

We identified patients who have ever received a critical illness diagnosis from January 1, 2000 to December 31, 2012 as the critical illness group. The critical illness was defined as the following disease: septicemia (ICD-9-CM codes 038), septic shock (ICD-9-CM codes 785.52), acute myocardial infarction (ICD-9-CM codes 410), hemorrhagic stroke (ICD-9-CM codes 430-432) and ischemic stroke (ICD-9-CM 433-438). The index date means the initial diagnostic date of critical illness. Subjects without any critical illness during the study period were randomly selected from the same database as the comparison group. Patients under 20 years old or who had a preexisting diagnosis of suicide attempt before the index date were excluded from the study. Patients in the critical illness and non-critical illness cohorts were matched at a ratio of 1:1 on the basis of a propensity score. The propensity score was calculated using the probability of the treatment assignment by using a logistic regression model and included the following baseline variables: year of index, age, and sex.

### 2.3. Outcome and comorbidities

The outcome of interest in this study was suicide (ICD-9-CM codes E950-E959), including liquid or solid poisoning (ICD-9-CM code E950), charcoal burning and poisoning by gases (ICD-9-CM code E952), hanging (ICD-9-CM code E953), cutting/piercing (ICD-9-CM code E956), jumping from high places (ICD-9-CM code E957), and others (ICD-9-CM codes E951, E954, E955, E958, and E959). All patients were followed from the index date to the occurrence of suicide, death, withdrawal from NHI program, or December 31, 2013, whichever came first. The chronic diseases under consideration included schizophrenic spectrum disorders, depressive disorder, alcohol-related illness, anxiety, mental disorders, insomnia, congestive heart failure, hypertension, diabetes mellitus, vascular diseases, Non-end-stage renal disease, end-stage renal disease, chronic obstructive pulmonary disease, malignancy, autoimmune diseases, and liver cirrhosis. The diagnostic accuracy of comorbidities based on ICD-9 codes has been examined in previous studies. Demographic data of monthly income, urbanization level, and occupation category were also collected.

### 2.4. Statistical analyses

The baseline demographic/clinical characteristics of the critical illness and the comparison cohorts were compared. A chi-square test and Student’s t-test were used to evaluate the distribution of category and continuous variables for an unmatched cohort. A standardized mean difference (SMD) of ≤0.10 indicates a negligible difference between the 2 matched cohorts. The incidence rates of suicide were calculated as per 10,000 person-years in both cohorts. The crude hazard ratios and 95% confidence intervals (CIs) of suicide occurrence were estimated using Cox proportional hazard regression. Multivariable models were further adjusted for depression, alcohol-related illness, anxiety, insomnia, and liver cirrhosis. We also examined the association between critical illness and suicide stratified by different subtypes of critical illness. The cumulative incidence of suicide during 12 years of follow-up was estimated for subjects with and without critical illness using Kaplan–Meier analysis, and assessed these differences using a log-rank test. We also considered death as a competing factor to estimate subhazard ratios and 95% CIs using the competing-risks regression models.^[13]^ The SAS 9.4 statistical package (SAS Institute Inc, NC, USA) was used for the statistical analyses. A 2-sided *P* value <0.05 indicated statistical significance.

## 3. Results

The demographics for the unmatched and matched cohorts were shown in Table 1. After matching, 48,651 patients in the critical illness cohort and 48,651 patients in the comparison cohort were enrolled in the study with similar distributions of age and gender by the propensity score. The majority of patients were aged 65 years or above, and males accounted for more than half of the patients in each cohort. The mean age was 65.7 ± 15.1 years in the critical illness cohort and 65.6 ± 14.9 in the comparison cohort. Compared with the comparison cohort, patients with critical illness had a significantly higher proportion of all the considered comorbidities (*P* < .001). The distribution of monthly income, urbanization level, and occupation category differed between the 2 cohorts (*P* < .001).

**Table 1:** Comparisons of demographic characteristics and comorbidities in patients with and without critical illness stratified by propensity score matching.

	Critical illness unmatched		*P*-value	Critical illness matched gender, age, and index year		SMD*
	No	Yes		No	Yes	
	(N = 622,882)	(N = 50,122)		(N = 48,651)	(N = 48,651)	
Gender			<0.001			
Women	314,339 (50.5)	21,175 (42.3)		20,046 (41.2)	20,774 (42.7)	0.03
Men	308,543 (49.5)	28,947 (57.8)		28,605 (50.8)	27,877 (57.3)	0.03
Age stratified			<0.001			
≤49	448,106 (71.9)	7917 (15.8)		7806 (16.0)	7917 (16.3)	0.006
50–64	118,584 (19.0)	12,619 (25.2)		12,544 (25.8)	12,619 (25.9)	0.004
65+	56,192 (9.02)	29,586 (59.0)		28,301 (58.2)	28,115 (57.8)	0.008
Age, mean ± SD†	42.3 ± 15.0	66.4 ± 15.4	<0.001	65.6 ± 14.9	65.7 ± 15.1	0.008
Monthly income‡			<0.001			
< 15,000	147,707 (23.7)	16,696 (33.3)		15,243 (31.3)	15,823 (32.5)	0.026
15,000–19,999	284,729 (45.7)	24,632 (49.1)		21,784 (44.8)	24,061 (49.5)	0.094
≥20,000	190,446 (30.6)	8794 (17.6)		11,624 (23.9)	8767 (18.0)	0.145
Urbanization level§			<0.001			
1 (highest)	194,224 (31.2)	12,310 (24.6)		13,758 (28.3)	11,947 (24.6)	0.09
2	184,525 (29.6)	13,296 (26.5)		13,065 (26.9)	12,917 (26.6)	0.007
3	114,801 (18.4)	8721 (17.4)		8189 (16.8)	8489 (17.5)	0.02
4 (lowest)	129,332 (20.8)	15,795 (31.5)		13,639 (28.0)	15,298 (31.4)	0.08
Occupation category∥			<0.001			
Office worker	357,642 (57.4)	17,660 (35.2)		19,879 (40.9)	17,293 (35.6)	0.11
Laborer	186,111 (29.9)	22,185 (44.3)		19,430 (33.9)	21,574 (44.3)	0.09
Other	79,129 (12.7)	10,277 (20.5)		9342 (19.2)	9784 (20.1)	0.02
Comorbidity						
Schizophrenia	4249 (0.68)	532 (1.06)	<0.001	209 (0.43)	527 (1.08)	0.08
Depression	18,806 (3.02)	3493 (6.97)	<0.001	2194 (4.51)	3408 (7.00)	0.11
Alcohol-related illness	15,325 (2.46)	3766 (7.51)	<0.001	1392 (2.86)	3740 (7.69)	0.22
Anxiety	65,092 (10.5)	11,060 (22.1)	<0.001	8796 (18.1)	10,738 (22.1)	0.10
Mental disorders	40,736 (6.54)	8546 (17.1)	<0.001	5660 (11.6)	8147 (16.8)	0.15
Insomnia	175,563 (28.2)	29,835 (59.5)	<0.001	21,967 (45.2)	28,809 (59.2)	0.28
Congestive heart failure	5093 (0.82)	6257 (12.5)	<0.001	2121 (4.36)	5914 (12.2)	0.29
Hypertension	92,870 (14.9)	34,719 (69.3)	<0.001	22,350 (45.9)	33,593 (69.1)	0.48
Diabetes mellitus	24,688 (3.96)	13,273 (26.5)	<0.001	5502 (11.3)	13,031 (26.8)	0.40
Vascular diseases	41,121 (6.60)	20,249 (40.4)	<0.001	11,556 (23.8)	19,493 (40.1)	0.36
Non-ESRD CKD	4292 (0.69)	4195 (8.37)	<0.001	1315 (2.70)	4043 (8.31)	0.25
ESRD	890 (0.14)	1250 (2.49)	<0.001	156 (0.32)	1233 (2.53)	0.19
COPD	30,602 (4.91)	12,563 (25.1)	<0.001	8530 (17.5)	11,835 (24.3)	0.17
Malignancy	8150 (1.31)	5184 (10.3)	<0.001	1951 (4.01)	5040 (10.4)	0.25
Autoimmune diseases	645 (0.10)	107 (0.21)	<0.001	69 (0.14)	107 (0.22)	0.02
Liver cirrhosis	82,833 (13.3)	12,975 (25.9)	<0.001	9645 (19.8)	12,724 (26.2)	0.15
Index year			<0.001			
2000	79,127 (12.7)	4233 (8.45)		4525 (9.30)	4151 (8.53)	0.03
2001	54,018 (8.67)	4360 (8.70)		3449 (7.09)	4282 (8.80)	0.06
2002	54,519 (8.75)	4512 (9.00)		3630 (7.46)	4413 (9.07)	0.06
2003	55,433 (8.90)	4164 (8.31)		3707 (7.62)	4056 (8.34)	0.03
2004	56,112 (9.01)	4570 (9.12)		4346 (8.93)	4441 (9.13)	0.007
2005	56,850 (9.13)	4589 (9.16)		4349 (8.94)	4453 (9.15)	0.007
2006	58,244 (9.35)	4619 (9.22)		4863 (10.0)	4491 (9.23)	0.03
2007	58,590 (9.41)	4618 (9.21)		5084 (10.5)	4452 (9.15)	0.04
2008	59,650 (9.58)	4692 (9.36)		5352 (11.0)	4520 (9.29)	0.06
2009	60,040 (9.64)	4837 (9.65)		5615 (11.5)	4652 (9.56)	0.06
2010	30,299 (4.86)	4928 (9.83)		3731 (7.67)	4740 (9.74)	0.07

Chi-square test.

COPD = chronic obstructive pulmonary disease, ESRD = end-stage renal disease.

* A standardized mean difference (SMD) of ≤0.10 indicates a negligible difference between the 2 cohorts.

† Student’s t-test.

‡ New Taiwan Dollar (NTD), 1 NTD is equal to 0.03 USD.

§ The urbanization level was divided by the population density of the residential area into 4 levels, level 1 was the most urbanized and level 4 was the least urbanized.

∥ Other occupation categories included those who were primarily retired, unemployed, and low-income populations.

Table 2 discloses the incidence and risk of suicide between the critical illness and comparison cohorts with propensity score. At the end of the study period, the overall incidence rates of suicide in critical illness cohort and comparison cohort were 1.58 and 0.41 per 10,000 person-years, respectively. After adjusting for confounding factors, critical illness cohort had a significantly increased risk of suicide than the comparison cohort (adjusted hazard ratio [aHR] = 2.98; 95% CI = 1.46–6.08). Table 2 also shows that patients with alcohol-related illness tended to have a higher risk for suicide (aHR = 3.86; 95% CI = 1.68–8.84).

**Table 2:** The incidences and risk factors for suicide with propensity score matching.

Variable	Event	PY	Rate*	Crude HR (95% CI)	Adjusted HR† (95% CI)
Critical illness					
No	11	269,117	0.41	1.00	1.00
Yes	29	183,740	1.58	3.81 (1.90–7.63)***	2.98 (1.46–6.08)**
Age group, years					
≤ 49	12	83,550	1.44	2.36 (0.96–5.77)	
50–64	8	131,196	0.61	1.00	1.00
65+	20	238,111	0.84	1.36 (0.60–3.10)	
Gender					
Women	21	191,925	1.09	1.51 (0.81–2.80)	
Men	19	260,932	0.73	1.00	1.00
Monthly income‡					
< 15,000	12	137,443	0.87	1.00	1.00
15,000 − 19,999	25	212,671	1.18	1.35 (0.68–2.69)	
≥ 20,000	3	102,743	0.29	0.34 (0.10–1.19)	
Urbanization level§					
1 (highest)	11	121,525	0.91	1.00	1.00
2	8	122,776	0.65	0.72 (0.29–1.79)	
3	10	77,610	1.29	1.42 (0.60–3.35)	
4 (lowest)	11	130,946	0.84	0.93 (0.40–2.14)	
Occupation category∥					
Office worker	15	176,848	0.85	1.00	1.00
Laborer	17	190,042	0.89	1.06 (0.53–2.11)	
Other	8	85,967	0.93	1.10 (0.47–2.59)	
Comorbidity					
Schizophrenia					
No	39	450,014	0.87	1.00	1.00
Yes	1	2843	3.52	3.97 (0.55–28.9)	
Depression					
No	35	429,934	0.81	1.00	1.00
Yes	5	22,923	2.18	2.67 (1.04–6.82)*	1.29 (0.45–3.71)
Alcohol-related illness					
No	32	435,845	0.73	1.00	1.00
Yes	8	17,012	4.70	6.34 (2.91–13.8)***	3.86 (1.68–8.84)**
Anxiety					
No	26	368,118	0.71	1.00	1.00
Yes	14	84,738	1.65	2.34 (1.22–4.50)*	1.56 (0.73–3.35)
Mental disorders					
No	37	397,714	0.93	1.00	1.00
Yes	3	55,143	0.54	0.58 (0.18–1.88)	
Insomnia					
No	13	234,801	0.55	1.00	1.00
Yes	27	218,055	1.24	2.25 (1.16–4.38)*	1.42 (0.69–2.90)
Congestive heart failure					
No	37	426,810	0.87	1.00	1.00
Yes	3	26,047	1.15	1.29 (0.40–4.21)	
Hypertension					
No	17	206,556	0.82	1.00	1.00
Yes	23	246,301	0.93	1.13 (0.60–2.12)	
Diabetes mellitus					
No	37	379,681	0.97	1.00	1.00
Yes	3	73,176	0.41	0.42 (0.13,1.35)	
Vascular diseases					
No	27	319,009	0.85	1.00	1.00
Yes	13	133,847	0.97	1.14 (0.59–2.22)	
Non-ESRD CKD					
No	38	436,371	0.87	1.00	1.00
Yes	2	16,486	1.21	1.35 (0.33–5.62)	
ESRD					
No	40	449,715	0.89	1.00	1.00
Yes	0	3142	0.00	-	
COPD					
No	30	372,507	0.81	1.00	1.00
Yes	10	80,350	1.24	1.53 (0.75–3.15)	
Malignancy					
No	39	437,350	0.89	1.00	1.00
Yes	1	15,507	0.64	0.68 (0.09–4.99)	
Autoimmune diseases					
No	40	452,279	0.88	1.00	1.00
Yes	0	578	0.00	-	
Liver cirrhosis					
No	24	356,119	0.67	1.00	1.00
Yes	16	96,738	1.65	2.45 (1.30–4.62)**	1.53 (0.77–3.02)

CI = confidence interval, COPD = chronic obstructive pulmonary disease, ESRD = end-stage renal disease, HR = hazard ratio; PY = person-years.

* Incidence rate per 10,000 person-years.

† Multivariable analysis included depression, alcohol-related illness, anxiety, insomnia, and liver cirrhosis.

‡ New Taiwan Dollar (NTD), 1 NTD is equal to 0.03 USD.

§ The urbanization level was divided by the population density of the residential area into 4 levels, level 1 was the most urbanized and level 4 was the least urbanized.

∥ Other occupation categories included those who were primarily retired, unemployed, and low-income populations.

*P < .05, **P < .01, ***P < .001.

Table 3 presents the stratified analyses with propensity score. The age-specific risk of suicide for critical illness cohort relative to comparison cohort was higher in patients aged under 65 years (aHR = 5.52, 95% CI = 1.56–19.50). When stratified by gender, the risk of suicide for critical illness cohort relative to comparison cohort was higher in both groups (aHR = 2.71, 95% CI = 1.03–7.14 in women and aHR = 3.17, 95% CI = 1.11–9.08 in men). Critical illness was significantly associated with an increased risk of suicide in the following sub-groups: patients with 15,000 to 19,999 monthly income, patients with lower urbanization levels, and laborer patients.

**Table 3:** Incidences and hazard ratios of suicide between individuals with and without critical illness stratified by demographics and comorbidity with propensity score matching.

	Critical illness							
	No			Yes				
Outcome	Event	PY	Rate*	Event	PY	Rate*	Crude HR (95% CI)	Adjusted HR† (95% CI)
Age group, years								
≤64	3	122,652	0.24	17	92,094	1.85	7.35 (2.15, 25.1)**	5.52 (1.56, 19.5)**
65+	8	146,465	0.55	12	91,645	1.31	2.42 (0.99, 5.93)	1.95 (0.79, 4.83)
p for interaction								0.31
Gender								
Women	6	111,080	0.54	15	80,845	1.86	3.46 (1.34, 8.92)*	2.71 (1.03, 7.14)*
Men	5	15,837	0.32	14	102,895	1.36	4.13 (1.49, 11.5)**	3.17 (1.11, 9.08)*
p for interaction								0.76
Monthly income‡								
<15,000	5	82,892	0.60	7	54,551	1.28	2.14 (0.68, 6.78)	1.65 (0.51, 5.31)
15,000–19,999	4	120,538	0.33	21	92,133	2.28	6.68 (2.29, 19.5)***	5.37 (1.81, 16.0)**
≥20,000	2	65,687	0.30	1	37,056	0.27	0.91 (0.08, 10.1)	0.97 (0.09, 11.1)
p for interaction								0.74
Urbanization level§								
1 + 2 (higher)	7	148,847	0.47	12	95,454	1.26	2.68 (1.05, 6.83)*	1.98 (0.75, 5.18)
3 + 4 (lower)	4	120,270	0.33	17	88,286	1.93	5.61 (1.89, 16.7)**	4.57 (1.51, 13.9)**
p for interaction								0.76
Occupation category∥								
Office worker	5	110,074	0.45	10	66,774	1.50	3.34 (1.14, 9.80)*	2.44 (0.80, 7.41)
Laborer	3	107,983	0.28	14	82,059	1.71	5.95 (1.71, 20.7)**	5.30 (1.50, 18.8)**
Other	3	51,060	0.59	5	34,907	1.43	2.37 (0.56, 9.93)	1.50 (0.34, 6.64)
p for interaction								0.89
Comorbidity¶								
No	2	77,287	0.26	1	11,976	0.83	3.55 (0.32, 39.2)	-
Yes	9	191,830	0.47	28	171,764	1.63	3.40 (1.60, 7.21)**	-
p for interaction								

CI = confidence interval; HR = hazard ratio; PY person-years.

* Incidence rate per 10,000 person-years.

† Multivariable analysis included depression, alcohol-related illness, anxiety, insomnia, and liver cirrhosis.

‡ New Taiwan Dollar (NTD), 1 NTD is equal to 0.03 USD.

§ The urbanization level was divided by the population density of the residential area into 4 levels, level 1 was the most urbanized and level 4 was the least urbanized.

∥ Other occupation categories included those who were primarily retired, unemployed, and low-income populations.

¶ Individuals with schizophrenia, depression, alcohol-related illness, anxiety, mental disorders, insomnia, congestive heart failure, hypertension, diabetes mellitus, vascular diseases, non-end-stage renal disease CKD, end-stage renal disease, chronic obstructive pulmonary disease, malignancy, autoimmune diseases, and liver cirrhosis were classified into the comorbidity group.

*P < .05,

** *P* < .01,

*** *P* < .001.

Table 4 shows the risk of suicide in patients with different subtypes of critical illness compared to those without critical illness without or with propensity score matching. In comparison to patients without critical illness, patients with septicemia, septic shock (aHR = 3.43, 95% CI = 1.52–7.74) and ischemic stroke (aHR = 3.04, 95% CI = 1.34–6.90) exhibited a significantly higher risk of suicide. Similar results were shown in the unmatched cohort.

**Table 4:** Comparisons of Incidence, and Hazard Ratios of suicide in different subtypes of critical illness compared to those without critical illness stratified by propensity score matching.

Variable	Event	PY	Rate*	Crude HR(95% CI)	Adjusted HR† (95% CI)
Without propensity score matching					
Critical illness					
None	291	4,026,154	0.72	1 (Reference)	1 (Reference)
Septicemia, septic shock	14	73,807	1.90	2.61 (1.53–4.47)***	2.53 (1.43–4.47)**
AMI	1	13,075	0.76	1.06 (0.15–7.53)	1.76 (0.24–13.0)
Hemorrhagic stroke	1	14,976	0.67	0.92 (0.13–6.57)	1.03 (0.14–7.40)
Ischemic stroke	13	84,175	0.76	2.13 (1.22–3.72)**	2.28 (1.25–4.17)**
With propensity score matching					
Critical illness					
None	11	269,117	0.41	1 (Reference)	1 (Reference)
Septicemia, septic shock	14	72,633	1.93	4.60 (2.09–10.2)***	3.43 (1.52–7.74)**
AMI	1	12,989	0.77	1.87 (0.24–14.5)	1.74 (0.22–13.5)
Hemorrhagic stroke	1	14,894	0.67	1.63 (0.21–12.6)	1.43 (0.18–11.1)
Ischemic stroke	13	83,224	1.56	3.80 (1.70–8.49)**	3.04 (1.34–6.90)**

CI = confidence interval, HR = hazard ratio, PY = person-years.

* Incidence rate per 10,000 person-years.

† Multivariable analysis included depression, alcohol-related illness, anxiety, insomnia, and liver cirrhosis.

** *P* < .01,

*** *P* < .001.

**Figure 1.**
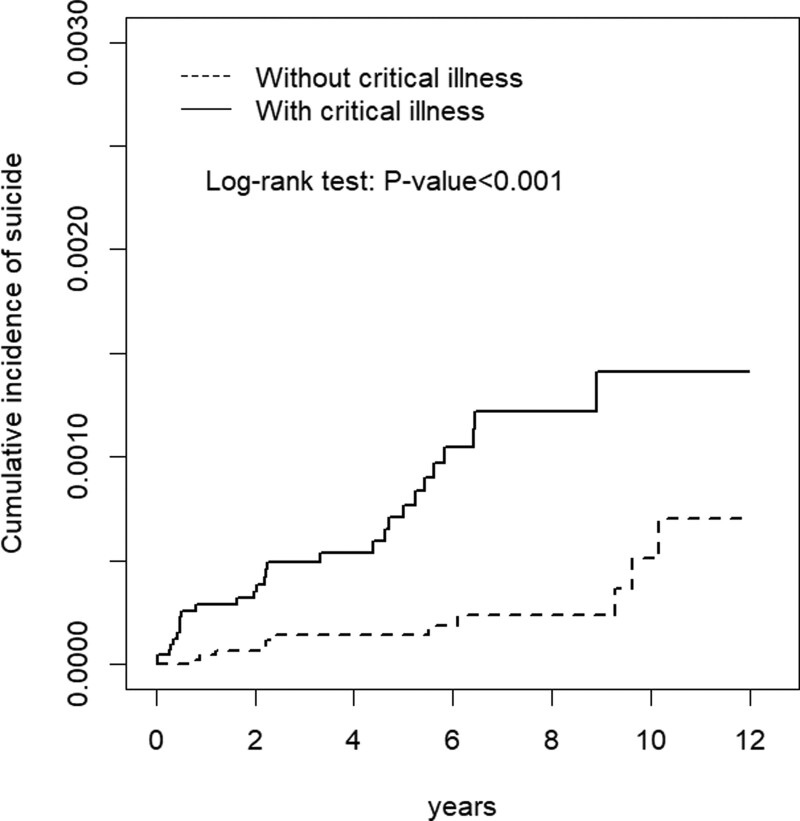
Cumulative incidence curves of suicide for groups with and without critical illness.

Kaplan–Meier survival analysis revealed that the cumulative incidence of suicide occurrence was significantly higher in the critical illness cohort than in the comparison cohort (log-rank test, *P* < .001) (Fig. 1).

Table 5 shows the subhazard rate of suicide for critical illness patients, with consideration of the competing risk of death. We found that the critical illness cohort still at a significantly higher risk of suicide than did the comparison cohort (aSHR = 2.98, 95% CI = 1.48–6.02).

**Table 5:** Critical illness cohort to non-critical illness cohort subhazard ratio (SHR) of suicide estimated using the competing-risks regression models with propensity score matching.

	Competing risks regression models	
	Critical illness	
	No	Yes
Suicide		
Crude SHR (95% CI)	1 (Reference)	3.81 (1.92–7.55)***
Adjusted SHR (95% CI)	1 (Reference)	2.98 (1.48–6.02)**

Crude SHR, relative sub hazard ratio; adjusted SHR: multivariable analysis included depression, alcohol-related illness, anxiety, insomnia, and liver cirrhosis (death was also added in the model to measure adjusted SHR).

SHR = subhazard ratio.

** *P* < .01,

*** *P* < .001.

## 4. Discussion

Using a large-scale database with propensity score matching, multivariable adjustment as well as competing risk method, the study demonstrates an association between critical illness and suicide. Moreover, sepsis/septic shock carried the highest risk among these critical illnesses.

It was possible to identify the mortality rate for suicide of about 13 per 100,000 in Taiwan.^[14]^ A very recent publication based on a Canadian data set had similar findings; this could be consistent with the control group having an abnormally low rate of suicide.^[15]^ Indeed, the large number of patients included is a major advantage of this study. Although some might criticize that the association might simply reflect the impact of comorbid illness since people with critical illness have more prevalence of medical comorbidity. The observation that sepsis/septic shock tended to be the highest risk of suicide attempt was surely a novel finding and deserved to be further highlighted.

The expected hypothesized association implies that further attention to patient surviving critical illness is mandatory. Additionally, the association appears to be higher with a diagnosis of sepsis/septic shock. Except for the impact of multiple comorbidities on the association, other possible biological links cannot be clearly identified primarily due to the methodology drawback of the investigation. Moreover, it is difficult to see a plausible biological mechanism for this, and confounding remains likely, making clinical application hard to propose clearly. The authors spend more time justifying this study by discussing potential pathophysiologic overlap between these conditions. People who have sepsis may develop an acute inflammatory response and the concept of systemic inflammation might be associated with suicide attempts despite some investigators might criticize that no sound scientific rationale is provided.^[16–18]^ It should be acknowledged that critically ill patients had more serious medical problems which might influence their suicide attempts, or they might receive it due to injury from their suicide attempts. Both are important confounders that should be considered. Finally, it could also be argued that choosing the critical illness cohort as newly diagnosed may artificially select for those who seek more treatment with more medical visits, making more incidence of suicide attempts in the study group. However, the association remained after incorporating the competing risk methods; additionally, sepsis is more susceptible to suicide attempts among these critically ill subjects, which might bring a new message and novel insights for the physicians caring for these specific groups. The appropriate position of this investigation in the literature might be reported with the above caveats as a justification for future research, and the detailed causal relationship and possible pathophysiological mechanism are strongly motivated.

## 5. Limitations

Despite the idea and data being novel, certain limitations in this study should be addressed. First, the study probably cannot change practice since the limitations of the study and lack of applicability of its findings.

Second, it appears that the disease, comorbidity, and outcomes were identified by the ICD-9 code in the record. Some might worry this may not be overly specific despite this algorithm have been validated previously.

Indeed, the study is based on an insurance database. Therefore, an underestimation of the outcome is likely since those who died shortly or immediately after suicide may not be admitted to the hospital. Finally, the retrospective data-based secondary analysis has certain inherent limitations and the ideal study is prospective with the serial examination, but of course, is tricky to perform.

## 6. Conclusion

An association between critical illness and suicide attempts was shown. Sepsis/septic shock was found to confer the highest risk in these specific populations.

## Author contributions

Wei-Syun Hu - Study concept and design, acquisition of data, analysis and interpretation, drafting of the manuscript, critical revision of the manuscript for important intellectual content, and study supervision. Cheng-Li Lin- acquisition of data, analysis, and interpretation.

## References

[1] Vincent JL. Critical care--where have we been and where are we going? Crit Care. 2013;17 Suppl 1(Suppl 1):S2. 23514264 10.1186/cc11500 PMC3603479

[2] Chelluri LP. Quality and performance improvement in critical care. Indian J Crit Care Med. 2008;12:67–76. 19742245 10.4103/0972-5229.42560 PMC2738304

[3] Weiss CH Amaral LA. Moving the science of quality improvement in critical care medicine forward. Am J Respir Crit Care Med. 2010;182:1461–2. 21159903 10.1164/rccm.201009-1483ED

[4] Stucky K Jutte JE Warren AM Jackson JC Merbitz N. A survey of psychology practice in critical-care settings. Rehabil Psychol. 2016;61:201–9. 27196862 10.1037/rep0000071

[5] Wade D Als N Bell V; POPPI investigators. Providing psychological support to people in intensive care: development and feasibility study of a nurse-led intervention to prevent acute stress and long-term morbidity. BMJ Open. 2018;8:e021083. 10.1136/bmjopen-2017-021083 PMC6059275 30037868

[6] Borrell-Carrió F Suchman AL Epstein RM. The biopsychosocial model 25 years later: principles, practice, and scientific inquiry. Ann Fam Med. 2004;2:576–82. 15576544 10.1370/afm.245 PMC1466742

[7] Lehman BJ David DM Gruber JA. Rethinking the biopsychosocial model of health: Understanding health as a dynamic system. Soc Personal Psychol Compass. 2017;11:e12328.

[8] Hatch R Young D Barber V Griffiths J Harrison DA Watkinson P. Anxiety, Depression and Post Traumatic Stress Disorder after critical illness: a UK-wide prospective cohort study. Crit Care. 2018;22:310. Vyas MV, . Association Between Stroke and Subsequent Risk of Suicide: A Systematic Review and Meta-Analysis. Stroke. 2021;52(4):1460-1464. 30466485 10.1186/s13054-018-2223-6 PMC6251214

[9] Liu CH Yeh MK Wang JH Weng SC Bai MY Chang JC. Acute coronary syndrome and suicide: a case-referent study. J Am Heart Assoc. 2016;5:e003998. 27927631 10.1161/JAHA.116.003998 PMC5210439

[10] Database NHIR. Taiwan. Available at: http://nhird.nhri.org.tw/en/index.html. [Access date May 10, 2017].

[11] Hu WS Lin CL. Association between cataract and risk of incident atrial fibrillation: a nationwide population-based retrospective cohort study. Mayo Clin Proc. 2017;92:370–5. 27902443 10.1016/j.mayocp.2016.08.019

[12] Hu WS Lin CL. Hemorrhoid is associated with increased risk of peripheral artery occlusive disease: a nationwide cohort study. J Epidemiol. 2017;27:574–7. 28268046 10.1016/j.je.2016.12.015 PMC5623014

[13] Fine JP Gray RJ. A proportional hazards model for the subdistribution of a competing risk. J Am Stat Assoc. 1999;94:496–509.

[14] Mortality rate for suicide in Taiwan from 2007 to 2019(per 100,000 population) Health, Pharma & Medtech›State of Health. Available at: https://www.statista.com/statistics/860970/taiwan-suicide-mortality-rate/.

[15] Fernando SM Qureshi D Sood MM. Suicide and self-harm in adult survivors of critical illness: population based cohort study. BMJ. 2021;373:n973. 33952509 10.1136/bmj.n973 PMC8097311

[16] Brundin L Bryleva EY Rajamani KT. Role of inflammation in suicide: from mechanisms to treatment. Neuropsychopharmacology. 2017;42:271–83. 27377015 10.1038/npp.2016.116 PMC5143480

[17] Bergmans RS Kelly KM Mezuk B. Inflammation as a unique marker of suicide ideation distinct from depression syndrome among U.S. adults. J Affect Disord. 2019;245:1052–60. 30699847 10.1016/j.jad.2018.11.046 PMC6448785

[18] Keaton SA Madaj ZB Heilman P. An inflammatory profile linked to increased suicide risk. J Affect Disord. 2019;247:57–65. 30654266 10.1016/j.jad.2018.12.100 PMC6860980

